# 1. Calcareous Uterine Fibroid. 2. Appendicitis*Reported to the Louisville Clinical Society.

**Published:** 1897-10

**Authors:** August Schachner

**Affiliations:** Louisville, Ky.; Demonstrator of Anatomy in the Louisville Medical College, Visiting Surgeon to the Louisville City Hospital, e c.


### 1. CALCAREOUS UTERINE FIBROID; 2. APPENDICITIS.*

By AUGUST SCHACHNER, M. D., LOUISVILLE, KY.

Demonstrator of Anatomy in the Louisville Medical College, Visiting Surgeon to the Louisville City Hospital, e c.

This tumor has one feature about it that is of some interest; it is one of those old fibrous tumors not usually seen. It was removed from a negro woman about sixty years of age. She came to me with this fibroid of the uterus and I removed it, taking away the entire uterus, by the abdominal method, leaving a small portion of the cervix behind. The interesting feature is the extensive calcareous degeneration which the tumor has undergone. We know that this calcareous formation occurs in these tumors when they grow old, and in old people. In several places the tumor is as hard as a rock.

Case 2. The second specimen is an appendix removed two weeks ago. The case illustrates two points, which, of course, have been mentioned quite frequently. The patient was a young German, whom I had operated upon a year ago for hernia and tumor of the testicle. He said he began complaining on the 13th of March with symptoms referable to the appendix. He was taken with pain in the right side of the abdomen, and I think sent for several doctors on Saturday afternoon. A physician in the immediate neighborhood saw him Sunday afternoon and administered one-half grain of morphine hypodermatically. He was given another dose of morphine on Sunday evening; he was still having considerable pain, though it was less severe, temperature slightly above 101 F., with marked rigidity of the right side.

I did not take charge of the case until twenty-four hours after that time; he still had a temperature of 101 degrees F., but not so much pain. Then the next day, twenty-four hours after I took charge of the case, I operated upon him; at the time of the operation the abdomen was very much distended; not much pain; temperature 101-2 degrees F. When I cut down to the appendix it was found very red, practically in a condition of necrosis; there was a perforation in the base of the appendix into which I could introduce my little finger;

---

*Reported to the Louisville Clinical Society.

about an ounce of thick pus surrounded the appendix. All the intestines were very red, distended, and covered with lymph. I took away the appendix, made a flap from the omentum and covered it thoroughly, and the patient made a good recovery. During the twenty-four hours after the operation, however, I had no hopes of the man making a recovery, and though the operation was performed about forty-eight hours after the onset of the trouble, I thought the case was hopeless for twenty-four hours thereafter.

As to the two points of interest: The first is, and I have made this statement before, that every case of appendicitis ought to be turned over to the surgeon immediately upon its recognition; i. e., it ought to be considered a surgical affection from the start. Physicians are too prone to treat these cases expectantly, and often state that all that is necessary is to keep the bowels open and the patient will get well promptly.

The second point is that this case very forcibly illustrates that if a case of appendicitis does not show marked signs of improvement within twenty-four hours after the onset of the trouble, they ought, as a rule, to be subjected to operation. We should regard appendicitis a surgical affection as strongly as we do strangulated hernia and other surgical affections. The wisdom of early operation in all cases of appendicitis has been proven so often that it is hardly necessary to dwell upon it at this time. It ought to be put down as a very general rule that if the patient does not show improvement in twenty-four hours after the onset of the trouble, he should be subjected to operation without further delay.

These two points are aptly illustrated by the case reported. The patient was operated upon forty-eight hours after the onset of the first symptoms, the appendix was in a necrotic state, with a large perforation at its base, with an ounce of pus surrounding the appendix, with the intestines red, distended, and covered with lymph. I believe if this man had gone twelve hours longer without on operation death would have been certain, whether operated upon or not. At the time of the operation he was in a state of general septic peritonitis.

DR. CARL WEIDNER: From my standpoint I have always been very conservative in cases of appendicitis: I believe in practicing conservatism in every case where it is possible. I will agree with the surgeon, however, that every case of appendicitis should be considered a surgical one. The question of time at which the operation should be performed I think is a different one entirely. I have seen two cases in the last few weeks, one a recurrent case that had been seen by one physician and a very excellent surgeon and operation had been proposed, but at the time they visited the patient he seemed so much better that the surgeon advised that the operation be postponed. A year afterwards the patient had a recurrence of the disease and I was called to see him. A surgeon was called in consultation and operated. The appendix was embedded in a thick mass of adhesions at the distal extremity, while the proximal one-half appeared to be in normal condition.

I saw a case in the city hospital a few days ago that is of some interest in connection with the necessity for operation. The patient had been complaining for three days of a little pain in the right side. He came to the hospital on the fourth day, and I made a diagnosis of appendicitis. There was some pain over the region of the appendix, but nothing typical. I made the diagnosis from the localized tenderness. The surgeon came in an hour afterwards and operated, finding the appendix bound down by firm adhesions.

DR. W. C. DUGAN: If there is any question in surgery that seems to me unsettled, it is appendicitis; *i. e.*, when we shall operate. I am like Dr. Schachner; if the patient does not show improvement inside of twenty-four hours, an operation should be performed. The most dangerous cases are those where there is no tumor; in other words, nature is not taking care of the condition. On the other hand, where there is a tumor nature has evidently walled in the abscess, and operation may be postponed for a few days. McBurney has laid great stress upon this point. When the symptoms are pronounced from the onset, where there is no tumor, and especially where the patient is subject to recurrent attacks, I would not hesitate to recommend immediate operation.

DR. LOUIS FRANK: In regard to Dr. Schachner's case of fibroid tumor of the uterus: I have seen several cases where there was extensive calcareous degeneration of old fibroid growths. We would see more of these cases if cases were more frequently operated upon. Calcareous degeneration takes place usually in the aged, where the tumors have been in existence for a long time, not attracting the attention of the surgeon, or not causing symptoms sufficiently pronounced to induce the patient to consult a surgeon. If more autopsies were made upon old women, especially among the negro population, I think we would find many of these calcareous tumors.

My views in regard to appendicitis are about the same as expressed by Doctors Schachner and Dugan. It is certainly a surgical disease, and the most important point is when shall we operate.

DR. AUGUST SCHACHNER: Operation for removal of the appendix, if done early, is not attended by much risk to the patient. We are all familiar with the heavy mortality in cases where the operation is delayed. I see nothing to be gained by waiting; if there is an abscess it should certainly be opened and drained just as we would treat an abscess elsewhere. Perforation takes place early in a great many cases, and without operation the usual termination is a fatal septic peritonitis.

